# A Case of the Use of Extracorporeal Carbon Dioxide Removal in a Patient With COVID-19 Acute Respiratory Distress Syndrome

**DOI:** 10.7759/cureus.24645

**Published:** 2022-05-01

**Authors:** Tarek R Firzli, Sunil Sathappan, Faisal Siddiqui

**Affiliations:** 1 Medical School, University of Nevada Reno School of Medicine, Reno, USA; 2 Critical Care Medicine, Veterans Affairs Sierra Nevada Health Care System, Reno, USA

**Keywords:** low tidal volume, invasive mechanical ventilation, mechanical ventilation, ecmo, respiratory acidosis, hypercapnia, cards, ards, covid-19, ecco2r

## Abstract

Acute respiratory distress syndrome (ARDS) is a severe complication of coronavirus disease 2019 (COVID-19) infection marked by increased fluid diffusely in alveolar spaces. The management of ARDS can be complicated by mechanical hyperinflation, and thus a mainstay of treatment has included low tidal volume mechanical ventilation. This, however, can lead to ventilation-associated hypercapnia, which may result in respiratory acidosis. COVID-19-associated ARDS (CARDs) has been described in the literature, and guidelines tend to mimic ARDS management. However, the heterogeneous nature of COVID-19 pulmonary disease with respect to dead space, compliance, and shunting could alter guidelines. As low tidal volume remains a cornerstone in CARDS management, hypercapnic acidosis remains a risk. An emerging technology, extracorporeal CO2 removal devices (ECCO2R), has been granted emergency use authorization by the FDA for the management of CARDS.

We present a 44-year-old male patient presenting positive for COVID-19. Following admission, the patient's oxygen status continually deteriorated and the patient went into acute respiratory distress, eventually requiring invasive mechanical ventilation. The patient became hypercapnic and acidotic due to low tidal volume ventilation. ECCO2R was used to manage the patient's hypercapnia, resulting in significant amelioration of his partial pressure of carbon dioxide (PCO2) and pH. The patient was eventually transferred to extracorporeal membrane oxygenation (ECMO) certified facility and survived after a prolonged hospital and rehabilitation course.

In the management of CARDS patients who require mechanical respiration, there are many unanswered questions as to the appropriate ventilation strategy. Current practice recommends low tidal volume ventilation, carrying, and increased risk of hypercapnic respiratory acidosis as occurred in our patient. We believe that ECCO2R may be an appropriate bridge between low tidal volume ventilation and ECMO to stabilize acid-base disturbances in ventilated patients.

## Introduction

Many severe acute respiratory syndrome coronavirus 2 (SARS-CoV-2) patients end up requiring supplemental oxygenation and invasive mechanical ventilation and can go on to develop acute respiratory distress syndrome (ARDS). Outcomes of patients requiring life support and invasive mechanical ventilation remain poor, with some studies estimating over 40% of patients who require invasive mechanical ventilation die in the hospital [1]. In mechanically ventilated patients, outcomes appear to improve when low tidal volumes, as well as pressures, are used [2], however, this has been shown to increase the risk of hypercapnia and hypercapnic acidosis [3]. Extracorporeal CO2 removal (ECCO2R) technology removes CO2 from the patient’s blood using a hollow filter cartridge and thus can be used to manage hypercapnia and resultant acidosis [4-5]. On April 22, 2020, the Food and Drug Administration (FDA) granted an emergency use authorization (EUA) for use of the Hemolung in critically ill coronavirus disease 2019 (COVID-19) patients suffering from ARDS [6]. Our institution had access to the ECCO2R device (Hemolung) for the purposes of use in COPD research. After the EUA was put in place, we were able to make it available to our patient population.

## Case presentation

**Figure 1:**
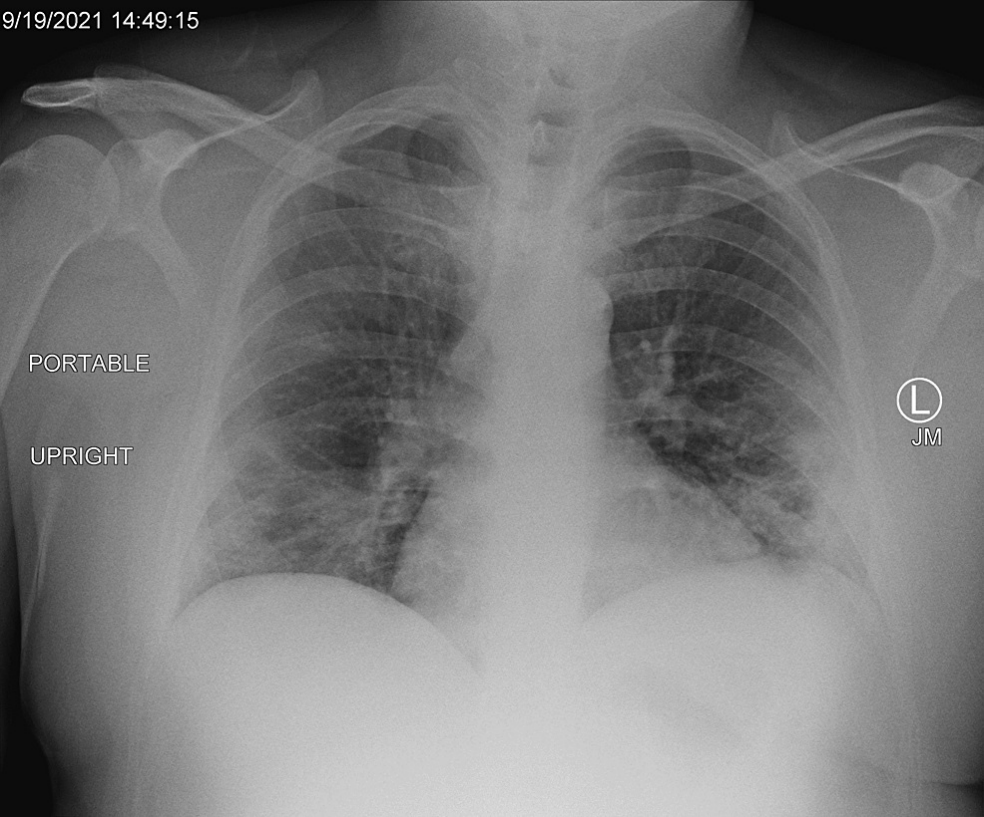
Admission chest radiograph (9/13/2021)

Our patient is a 44-year-old, male, unvaccinated against COVID-19 who presented with intermittent cough, shortness of breath, fevers, nausea, and diarrhea, which onset one week prior to admission. His admission vitals showed a temperature of 98.3 °F, pulse of 81 bpm, respiratory rate of 16 breaths/min, and blood pressure of 112/72 mmHg. His admission labs showed a positive COVID-19 polymerase chain reaction (PCR) test, serum lactate dehydrogenase of 373 IU/L (N: 83-189), creatinine phosphokinase of 354 IU/L (N: 0-243), international normalized ratio (INR) of 1.2, fibrinogen greater than 676 mg/dL (N: 276-471), D-dimer of 333 ng/mL (N<=230), ferritin of 977 ng/mL (N: 22-322), erythrocyte sedimentation rate of 17 mm/h (N: 0-20), and lactic acid of 1.0 mmol/L (N: 0.6-2.7) (Table 1). His liver enzymes were mildly elevated with normal renal function, troponins, and electrolytes. His admission chest radiograph showed scattered patchy ground-glass opacities consistent with viral pneumonitis (Figure 1).

**Table 1:** Labs on admission

Lab Test	Lab Value
Lactate Dehydrogenase	373 IU/L (N: 83-189)
Creatinine Phosphokinase	354 IU/L (N: 0-243)
INR	1.2
Fibrinogen	>676 mg/dL (N: 267-471)
D-Dimer	333 ng/mL (N<= 230)
Ferritin	977 ng/mL (N: 22-322)
Erythrocyte Sedimentation Rate	17 mm/h (N: 0-20)
Lactic Acid	1.0 mmol/L (N: 0.6-2.7)

The patient was initially admitted to the medical floor and required supplemental oxygenation to keep his oxygen saturation above 90%. During his stay, his oxygen requirements continued to increase, and he was placed on noninvasive ventilation (BiPAP). He received treatment with remdesivir and dexamethasone followed by tocilizumab.

**Figure 2:**
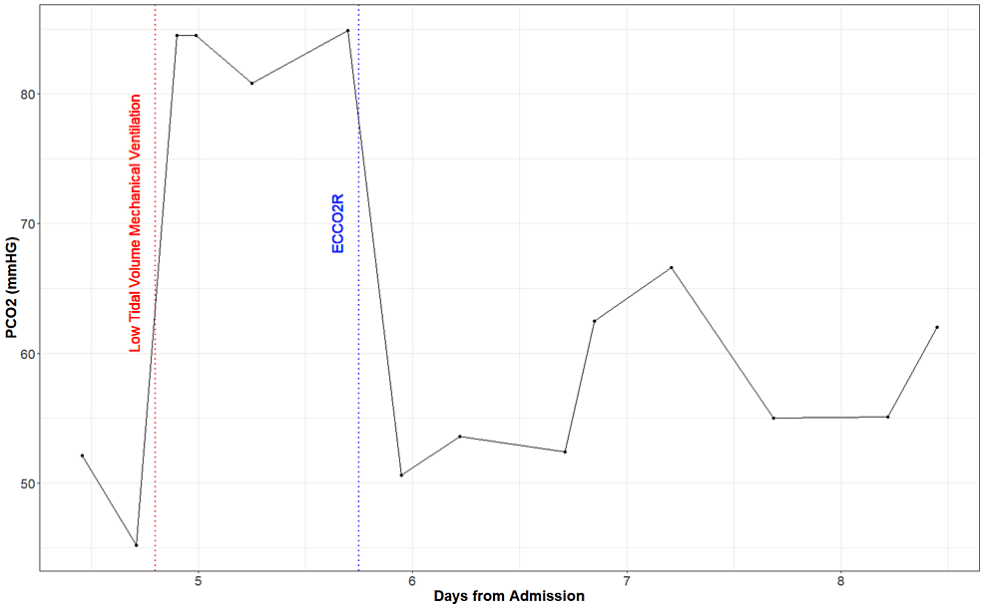
Partial pressure of carbon dioxide (PCO2) from first arterial blood gas measurement (time=0) until the patient left the facility

**Figure 3:**
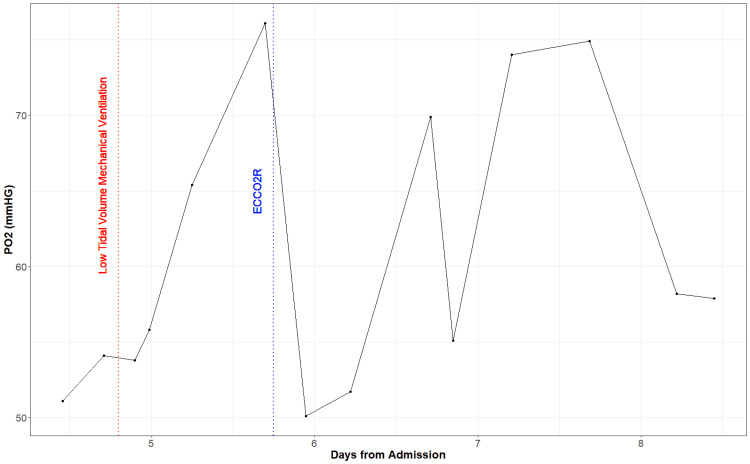
Partial pressure of oxygen (PaO2) measurements from the day of admission (arterial blood gas (ABG) measurements only available on Day 4)

**Figure 4:**
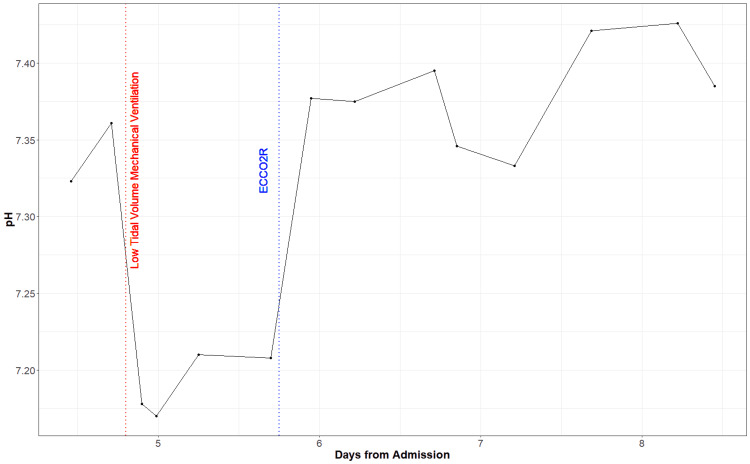
pH measurements from the day of admission (arterial blood gas (ABG) measurements only available on Day 4)

**Figure 5:**
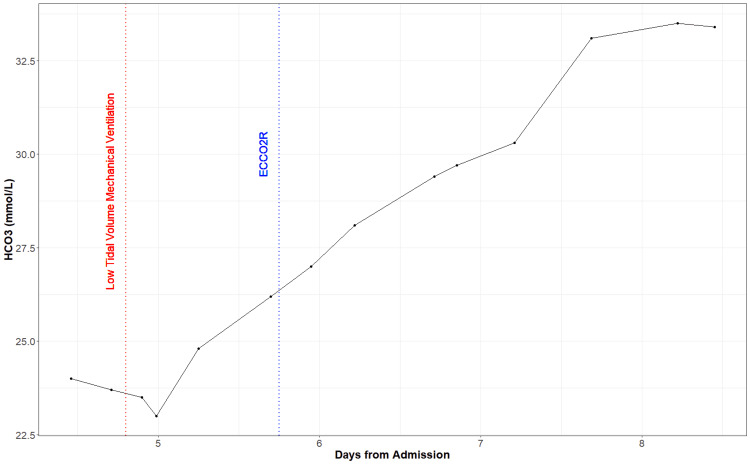
Bicarbonate (HCO3) measurements from the day of admission (arterial blood gas (ABG) measurements only available on Day 4)

The patient’s oxygenation continued to worsen, and he was intubated for mechanical ventilation on Day 4. His oxygenation status did not improve even after the neuromuscular blockade, and as we pursued a low tidal volume ventilation strategy, the patient's arterial concentration of CO2 (PaCO2) continued to rise. From the time of intubation, the patient’s PaCO2 was markedly increased (mean=83.68 mmHg) (Figure2), partial pressure of oxygen (PaO2) was decreased (mean=62.78 mmHg) (Figure 3), pH was markedly decreased (mean=7.19) (Figure 4), and bicarbonate (HCO3) was low to normal (mean=24.38 mmol/L) (Figure 5). We decided to initiate an ECCO2R device (Hemolung) to improve the patient's arterial blood gases and hemodynamics on Day 5. We used a 15.5 Fr Hemolung catheter in the right femoral vein. On the first day, the blood flow was 340-350ml/min, and sweep gas flow was started at 4L/min and ended at 5L/min. On the second day, the blood flow was 360-400 ml/min while the sweep gas flow was reduced from 5L/min to 4L/min. The blood flow settings remained the same on the following days but sweep gas was gradually reduced down to 0L/min when the patient was eventually weaned from the Hemolung. After starting this device, patient’s PaCO2 dropped (mean=57.23 mmHg) (Figure 2), PaO2 varied but remained somewhat low (mean=61.48 mmHg) (Figure 3), pH improved (mean=7.38) (Figure 4), and HCO3 increased steadily (mean=30.56 mmol/L) (Figure 5). At the end of Day 7, the patient was being prepped for transfer and demonstrated much more variability in hemodynamic parameters. The patient was eventually transferred to an ECMO-certified medical center and underwent veno-venous (VV) ECMO. After a long period of intensive care intervention, he was eventually discharged to a rehabilitation facility.

## Discussion

In the management of ARDS, low tidal volume ventilation strategies have been the cornerstone for critical care treatment [2,7-8]. The benefit of this strategy is to decrease hyperinflation and subsequent lung injury and has thus been utilized in mechanically ventilated COPD and ARDS patients. However, in COVID-19-associated ARDS, this strategy is still unclear. In general, there is some consensus, in fact, that ARDS secondary to COVID-19 does not behave in a similar fashion to ARDS secondary to other sources in increased dead space and higher compliance in COVID-19 patients [9-10], which has led to the classification of CARDS [11]. This syndrome is marked by heterogeneous features and could differ by patient or even viral strain. It has thus been suggested that different patterns of CARDS may exist, and each could require different treatment modalities [12]. NIH guidelines have suggested tidal volume values should range from 4-8 ccs [13] while Surviving Sepsis Campaign panels have recommended similar treatment for CARDS and non-COVID-19-related ARDS patients [14]. Management of ventilation strategies depends on several factors including compliance, dead space, ventilation/perfusion ratios, and oxygenation, all of which are still being studied in COVID-19 patients. Irrespective of these potential differences, low tidal volume ventilation is clinically utilized in CARDS, and thus places patients at risk for hypercapnia and respiratory acidosis.

ECCO2R has demonstrated efficacy as a means of carbon dioxide (CO2) removal for patients who are otherwise unable to adequately oxygenate, whether it be a result of ARDS, COPD, or some other etiology [15]. A study by Pisani et al. notes that the device, when coupled with non-invasive ventilation (NIV), serves as a less invasive and more effective alternative to invasive mechanical ventilation (IMV) among patients experiencing severe respiratory acidosis [16]. A number of case reports show that ECCO2R can also aid the early and prompt removal of CO2 in intubated patients, potentially mitigating the high mortality associated with prolonged intubation [17-18]. Another report highlights utility among a patient experiencing CARDS and undergoing mechanical ventilation, with ECCO2R aiding in the successful correction of respiratory acidosis whilst enabling ultra-low tidal volumes that limited the likelihood of ventilator-associated morbidity and mortality [19]. Other strategies do exist to handle respiratory acidosis, namely, the use of bicarbonate infusion. Although generally reserved for metabolic acidosis, the administration of bicarbonate to treat respiratory acidemia is prevalent in ICUs. Bicarbonate infusion for respiratory acidosis, however, remains a controversial strategy as there is a lack of clinical evidence supporting its use [20]. Some experts even argue against its use given the potential risks associated with its administration. These factors led the treating team to prefer the use of ECCO2R to bicarbonate administration.

In our case, the patient was initiated on low tidal volume ventilation, however, he began to experience extreme hypercapnia, on average over 80 mmHg. This led to acid-base disturbances, with his pH averaging 7.19, which was not adequately compensated for by bicarbonate production which remained low-normal. Overall, this patient demonstrated hemodynamic compromise secondary to his acid-base status, and the decision was made to employ the Hemolung extracorporeal carbon dioxide removal device to assist with CO2 removal as a bridge between low tidal volume ventilation alone and extracorporeal membrane oxygenation (ECMO). Eventually, the patient was weaned from ECCO2R and transferred to a facility that could provide the patient with ECMO. The utilization of ECCO2R in hypercapnic respiratory failure secondary to low tidal volume ventilation was shown to be a useful strategy in this patient. We feel that more research is required to confirm the benefit of low tidal volume ventilation paired with extracorporeal CO2 removal in such patient populations. Furthermore, as CARDS may have a heterogeneous pattern, additional research is important to establish different CARDS phenotypes that could aid in clinical decision-making. 

## Conclusions

The strategy of low tidal volume, or even ultra-low tidal volume, approaches have been widely recommended by the NIH and SSC in ARDS patients owing to documented mortality benefits. This has become a mainstay of treatment in ARDS, and as COVID-19-associated acute respiratory distress syndrome became described, low tidal volume strategies were recommended. No matter the reason for utilizing low tidal volume ventilation, and although it has been shown to effectively reduce mortality in certain conditions, it is not without risks. One particularly pertinent risk is hypercapnic respiratory acidosis, caused by hypoventilation of CO2 secondary to the ventilator settings themselves. Extracorporeal carbon dioxide removal devices were developed as a treatment for COPD exacerbation where it is extremely important to maintain low tidal volumes to prevent lung injury due to hyperinflation. These devices can provide relatively quick reversal of hypercapnia in mechanically ventilated patients and stabilize or correct worsening acid-base status in these patients. In our patient, low tidal volumes were used when CARDS began to develop, and he was unable to adequately oxygenate. This, however, led to hypercapnic respiratory acidosis. The use of ECCO2R stabilized the acid-base disturbance in our patient, providing time to transfer him to an ECMO facility. In mechanically ventilated COVID-19 patients with ARDS, we believe this to be a useful tool for clinicians in managing hypercapnia.
